# What matters when asking, “what matters to you?” — perceptions and experiences of health care providers on involving older people in transitional care

**DOI:** 10.1186/s12913-020-05150-4

**Published:** 2020-04-16

**Authors:** Cecilie Fromholt Olsen, Jonas Debesay, Astrid Bergland, Asta Bye, Anne G. Langaas

**Affiliations:** 1 Department of Physiotherapy, OsloMet - Oslo Metropolitan University, Oslo, Norway; 2 Department of Nursing and Health Promotion, OsloMet – Oslo Metropolitan University, Oslo, Norway; 3 Regional Advisory Unit in Palliative Care, Department of Oncology, Oslo University Hospital, Oslo, Norway

**Keywords:** Aging, Older people, Chronic disease, Transitional care, Patient-centered care, Person-centered care, Quality improvement, Health care providers, “What matters”

## Abstract

**Background:**

Transitional care for older chronically ill people is an important area for healthcare quality improvement. A central goal is to involve older people more in transitional care and make care more patient-centered. Recently, asking, “What matters to you?” (WMTY) has become a popular way of approaching the implementation of patient-centered care. The aim of this study was to explore health care providers’ perceptions and experiences regarding the question of WMTY in the context of improving transitional care for older, chronically ill persons.

**Methods:**

The data comprise semi-structured individual interviews with 20 health care providers (HCPs) who took part in a Norwegian quality improvement collaborative, three key informant interviews, and observations of meetings in the quality improvement collaborative. We used a thematic analysis approach.

**Results:**

Three interrelated themes emerged from the analysis: WMTY is a complex process that needs to be framed competently; framing WMTY as a functional approach; and framing WMTY as a relational approach. There was a tension between the functional and the relational approach. This tension seemed to be based in different understandings of the purpose of asking the WMTY question and the responsibility that comes with asking it.

**Conclusions:**

WMTY may appear as a simple question, but using it in everyday practice is a complex process, which requires professional competence. When seen in terms of a patient-centered goal process, the challenge of competently eliciting older people’s personal goals and transferring these goals into professional action becomes evident. An important factor seems to be how HCPs regard the limits of their responsibility in relation to giving care within the larger frame of the patient’s life project. Factors in the organizational and political context also seem to influence substantially how HCPs approach older patients with the WMTY question.

## Background

With an aging population and increase in chronic health problems among older people [1], stakeholders have shifted their focus to transitional care [2]. Transitional care is a broad term for care interventions that promote the safe and timely transfer of patients between levels of care and across settings, such as from hospital to home or other care settings in the community [3, 4]. Improving the quality of transitional care for older people is a high priority in many countries, including Norway and ultimately aims at reducing costly hospital readmissions and achieving an overreaching political goal of older people living in their own homes for as long as possible [5]. Increased pressure to discharge people from hospitals quickly has raised concerns that older patients are being prematurely discharged and will subsequently be readmitted [6]. The transition between levels of the health care system and the period subsequent to hospital discharge is especially vulnerable and stressful for older, chronically ill patients [7].

One important aspect of improving the transitional care experience is to make transitional care more patient-centered. Previous research on patient involvement in transitional care has revealed that older patients experience a lack of participation during transitions [8–11]. Transitional care is also characterized by the challenges of coordinating care across organizational and disciplinary boundaries, managing conflicting goals of care, which may be stressful for health care providers (HCPs) [12–14]. Dyrstad et al. [8] found that the focus on efficiency and time constraints affected patient involvement negatively, and practices were often task-oriented instead of patient-oriented. HCPs perceived older chronically ill patients as being too frail to be involved, but they were also uncomfortable with involving older patients because of the fear of unrealistic goals [8, 15].

In the current study, we followed the work of HCPs who took part in a Norwegian national quality improvement initiative that was based on collaborative quality improvement. Establishment of a quality improvement collaborative (QIC) is a strategy for quality improvement involving multidisciplinary teams from various health care settings coming together over several months to improve their provision of care [16]. Making transitional care more holistic and patient-centered was a central goal for this particular QIC, and the question “What matters to you?” (WMTY) was the chosen way of approaching patient-centeredness. This prompted us to explore more in-depth the HCPs’ perceptions and experiences regarding the WMTY question.

### The origin of “What matters to you?”

In an article in the *New England Journal of Medicine* in 2012, Barry and Edgeman-Levitan introduced the concept of asking “What matters to you?” in addition to “What is the matter with you?” as a way of implementing patient-centered care and shared decision making [17]. Since then, the Institute for Healthcare Improvement (IHI) [18] has been largely responsible for spreading the question as a slogan and symbol for patient-centered care. WMTY has been spread to several countries across the world and is characterized as a global movement. In Norway, the WMTY question has become part of a political rhetoric to improve quality in health care. Along with slogans such as, “no decision about me, without me” and creating “the patient’s health care system,” WMTY is used to promote patient-centered care [19, 20]. According to the IHI [18], WMTY is about compassion, seeing the person behind the patient, and realizing in a more profound way what matters to patients both on a system level and individual one. Furthermore, WMTY is presented as a tool for personal goal setting, the ideal being that all patients should have the opportunity to set and strive for personal and clinical goals. It is central that the patients’ personal goals should guide clinical decision making [18]. Basing practice on WMTY also has the potential to improve joy at work [21–23]. However, despite the growing use and popularity of the WMTY question, little research has been done on HCPs perceptions and experiences regarding its use in everyday health care practice. Most of what has been published about asking WMTY is in the form of policy and opinions.

### Conceptual framework

WMTY is based on the concepts of person-centered and patient-centered care (PCC)^1^ These are multifaceted concepts that several concept analyses have set out to define [24]. The commonalities of these concepts are that they constitute new models of care that can be conceptualized as responses to a biomedical paternalistic health care model in which the patient has a passive, dependent role and the HCP is the decision-making expert. A central belief is that patients gain more power over their own lives and decisions of care through increased involvement [24, 25].

Goal orientation is generally recognized as a central feature of health care and PCC, and patient-centered goal setting should be aimed at multiple dimensions of an older person’s life [25–27]. Goals are here understood as a desired future state of affairs towards which effort and resources are directed [28]. In transitional care for the older chronically ill, fragmentation and conflicting care goals represent a core challenge [14, 29]. Both in case of conflicting goals or complementary goals that compete for resources, a competent prioritization of goals is called for [28, 30, 31]. In health care, this is often done by creating a goal hierarchy, where the highest level goal is broken down into subgoals and tasks [28, 30, 31].

A recent concept analysis revealed many shared attributes of PCC, but there is a distinction between the concepts when it comes to the overreaching goal. The shared attributes of the two concepts are empathy, respect, engagement, relationship, communication, shared decision making, holistic focus, individualized focus, and coordinated care. However, the goal of person-centered care is a meaningful life, while the goal of patient-centered care is a functional life [24]. In the context of quality improvement, patient-centeredness is one of six essential features of quality in health care and is defined as “care that is respectful of and responsive to individual patient preferences, needs, and values and [ensures] that patient values guide all clinical decisions” [32]. In patient-centered care, the focus is to include a more holistic view of the patient and their wider social and cultural background. Person-centered care is grounded in the concept of personhood and focuses more on how people are recognized as unique individuals rather than patients, thus going beyond the patient role [25]. This places health care into the larger frame of the person and their “life project” [33]. However, regardless of these differences, both person-and patient-centered care are complex interventions, and their implementation into practice has proven to be difficult [26, 34]. Previous research shows that PCC may be adopted by practitioners on a principal level, but they do not necessarily become embedded in everyday clinical practice [34, 35].

PCC practices should be understood based on the complex interplay between many factors at the individual and system level [36, 37]. In line with this understanding, previous research has shown that the barriers for PCC implementation involve factors both at the individual, organizational, and political levels [10, 26, 34]. Different components of PCC may be differently constructed and valued by different stakeholders and for different reasons. At the individual level, previous research has identified provider–patient communication and professional competence as key to achieving PCC [38, 39]. Mik-Meyer [37] emphasized that an increased focus on personalization in healthcare policy and practice can create new challenges for HCPs and patients, who must take on new roles and responsibilities [37]. According to Epstein and Hundert [40], professional competence is “the habitual and judicious use of communication, knowledge, technical skills, clinical reasoning, emotions, values and reflection in daily practice for the benefit of the individual and the community being served” (p.226). Professional competence can be divided into relational competence and action competence [41, 42]. Relational competence in PCC models focus on the ability to listen, have empathy, be reflective, and know the self [24, 38]. Here, action competence is generally understood as instrumental skills and knowledge and is related to practical tasks and standardized procedures that providers perform as part of their work with patients [43, 44]. These two competences are knit together and complementary in encounters between providers and patients. Consistent with this, Berntsen et al. [14] maintain that, in a patient-centered goal setting process, HCPs need the competence to elicit goals sensitively and to be able to translate personal goals into realistic and manageable professional goals. Dilemmas and tensions in practice often occur pertaining to having to fulfill professional tasks while maintaining the relationship and respecting the patient’s needs, preferences and goals [14, 41, 43, 45]. At the system level, organizational factors play an important role. Factors such as a lack of resources and conflicting organizational goals are viewed as barriers, while organizations that incorporate a general person-centered culture are viewed as facilitating [26, 34, 38]. This is perhaps particularly salient for transitional care, which operates at the intersection of several different organizational cultures. Berntsen et al. [14] propose that putting the patients’ goal at the top of the prioritization list can solve the many tensions pertaining to conflicting goals in healthcare. Hence, asking WMTY represents a promising solution for the prioritization of professional goals and may serve as a guiding principle [14] and a “bridge” between the different professionals and settings in transitional care for older people.

Based on these conceptual underpinnings, the aim of the current article is to explore HCPs’ perceptions and experiences of the use of WMTY. We believe that greater insight into these features will benefit all stakeholders in the field of user involvement and PCC, especially those interested in transitional care for older chronically ill persons.

## Method

The current study is a qualitative study comprising observations of 22 QIC meetings and individual semi-structured interviews with 20 HCPs and three key informants. It was part of a larger study aimed at exploring the development of good patient pathways for older chronically ill people. A social constructivist approach informed the study [46].

### Context

The study took place in a large municipality in Norway. The QIC was assembled as part of a larger national quality improvement initiative in Norway, and it followed a model of collaborative learning known as *The Breakthrough Series,* developed by the IHI [16, 47]. The current QIC met over a period of 18 months and brought together multidisciplinary teams from one hospital and multiple primary care settings (institutional care and home care). The approximately 90 participants of the QIC met at four 2-day gatherings, called learning sessions, which were three, six, and nine months apart. In between the four learning sessions, the participants worked in their local teams to implement the improvement measures discussed at learning sessions. Team leader meetings were held regularly. During the learning sessions, the PCC aspect of transitional care was introduced to the participants through the symbolic question of WMTY, which, in Norwegian, translates to “What is important to you?” The participants were also introduced to a tool called the “WMTY questionnaire,” which is based on the Patient Specific Functional Scale, a standardized questionnaire used for personal goal setting [48]. This was presented as a tool to facilitate and measure the use of WMTY in practice. It should be mentioned, that the QIC was not the only promoter of the WMTY question to the participants, as this was a general trend consistent with current healthcare quality discourse in Norway.

### Recruitment

The inclusion criteria for taking part in the present study were to be a HCP, involved in the activities of the relevant QIC, and thus involved in transitional care for older, chronically ill persons. The QIC administrators gave permission and access to the field. Prior to the first learning session, an email containing project information and written consent forms was sent to the QIC participants. At the learning sessions, consent forms were collected, and the participants were informed orally in plenum about the research project. The QIC administrators assisted in gaining access to observe at the local team meetings after the participants had given their initial consent for observation, and the researcher worked to retain this access through the research period. For the individual interviews, a purposively collected sample was chosen [49]. We wanted to interview as many improvement team leaders as possible, believing that they would be particularly informed regarding the topics of interest. We also aimed to reach participants from a variety of professional backgrounds and settings to reflect the QIC and the transitional care context. Recruitment continued throughout the collaborative period, as new participants joined the improvement teams. We believed we had enough information power [50] at 17 HCP interviews, but to be sure, we conducted another three interviews.

### Data collection

Data were collected over the course of the QIC period of 18 months from 2017 to 2019. The first author conducted all the semi-structured interviews and observations. A thematic interview guide was used (see Additional file 1). An open approach was chosen to give room for the participants to talk about what they were working on in their respective improvement teams. Whilst remaining within the core themes, the interview questions were adapted along the course of the QIC’s work according to their progression. For example, initial questions about patient involvement changed into concrete questions about WMTY as it became clear that patient involvement was approached through this question. The researcher inquired about the participants’ general views on asking WMTY and their experiences of using the question in practice. The WMTY questionnaire was shown during most of the interviews as a reminder of what had been discussed during the QIC meetings. All interviews were audio recorded and transcribed verbatim. The interviews were conducted in the participants’ respective workplaces. They lasted from one to one and a half hours.

The researcher observed at six local improvement team meetings, 13 team leader meetings and three administrators meetings. She also attended the four learning sessions to understand the context of the QIC. Apart from learning sessions, which took place in conference venues, the QIC meetings were held in the respective workplace environments and in the administrators’ localities. Meetings could last from one to 7 hours, depending on the type of meeting and topics discussed. During observations, the researcher sat together with the participants, she engaged in small talk, but did not partake in the participants’ discussions. Features of the setting, actions performed, and the tools used were written down. The researcher also wrote down what was being said when of interest to the topic of WMTY, patient-centeredness, user involvement, and care pathway development. Sometimes, statements were captured near verbatim, and some of these statements were used as quotes in the results section. After the observation, the researcher wrote down memos concerning contextual factors and reflections regarding the observations. The participants knew the professional background of the researcher. Because the researcher met with the participants frequently over a period, a relationship was built with several of the participants.

### Participants

The 20 HCPs who participated in the individual interviews had a mean age of 43.9 years with a range of 29–59 years. There were four men and 16 women. Most of the participants (*n* = 12) were nurses who held different positions: five worked with professional development or coordination in addition to working front-line with patients, four worked only front-line with patients, and three held full manager positions. Five of the participants were physiotherapists, one was an occupational therapist, one was a doctor, and one was an assistant nurse. All of these worked front-line. Thirteen of the participants worked in the home care sector, five worked in short-term institutional care (intermediate, rehabilitative and acute care), and two worked in a hospital. The participants had worked in their current positions between 6 months and 17 years with a mean time of 5.1 years. The mean number of years of education after high school was 5.1 years with a range of 3–10 years. Seven of the participants were leaders of their local improvement team in the QIC. All the interviewed participants were chosen by their respective superiors to take part in the QIC activities. One of the nurses was not a member of a particular improvement team, but took part in one of the team’s improvement efforts related to WMTY. In addition to HCP interviews, three key informants who were leaders or administrators in the QIC were interviewed to gain understanding of contextual factors. We wanted to understand the underlying ideas and perceptions which formed the basis of the QIC such as the WMTY question. We also enquired about the purpose and content of learning sessions and meetings. The same thematic guide was used, but the questions were formulated to suit the participants’ position in the QIC.

Team leaders and participants from the local improvement teams and persons with expertise in relevant areas such as electronic health record systems attended the QIC meetings. At the improvement team leader meetings 10–15 participants were usually present, at the local improvement team meetings 3–10 participants were usually present. At the three administrators meetings, 5–10 participants were present.

### Data analysis

The first author transcribed 20 of the interviews; an assistant transcribed three interviews. The data were structured using NVivo Pro 12 software (NVivo qualitative data analysis software Melbourne, Australia: QSR International Pty Ltd., 2018) and analyzed using thematic analysis as described by Braun and Clarke [51, 52]. Memos covering analytical reflections were written during the process. An analytic matrix with the extracts from the interviews concerning PCC and WMTY was made in a table. Based on a preunderstanding of the implementation of PCC as a difficult and complex endeavor and a certain skepticism toward the WMTY question, we asked the following analytical questions: What tensions and/or dilemmas become apparent when HCPs talk about their perceptions and experiences regarding the WMTY question? This included looking at how the HCPs talked about using the question in practice, what concerns they had about it, and how they understood the purpose of WMTY. Inspired by previous research, we also asked what new roles the WMTY question could imply for HCPs, and how they perceived their responsibility in relation to the question. Finally, we asked how contextual factors might have influenced the HCPs’ perceptions and experiences regarding WMTY.

One analytical challenge, especially regarding contextualizing the findings, was that the participants from the QIC came from diverse health care settings, which would arguably create different conditions for PCC at the micro level. When analyzing the data, however, there were no definite patterns pointing to certain settings being different from others concerning the WMTY question. Hence, we chose to analyze the data based on commonalities across settings rather than local variations.

Preliminary coding and themes concerning WMTY, user involvement, and PCC were developed from the data without a particular theoretical framework in mind. However, the final themes were influenced by recent concept analyses of PCC and the view that PCC is a complex phenomenon operating at both the system- and individual level. The concepts of professional competence and patient-centered goal setting came to mind toward the end of the analysis because it provided useful insight into the tension between the two approaches to WMTY. Our use of concepts in the discussion resembles that of theoretical bricolage [53] because a diverse range of concepts and viewpoints from previous research on PCC were used to highlight the primarily inductively developed themes.

The first author performed the initial analysis, and the other authors commented critically to challenge and elaborate on the analysis. Regarding the researchers’ positions, it should be noted that all authors have a background as both professionals and researchers in the fields of physiotherapy, nursing, dietetics and social anthropology respectively. This allowed for a breadth in perspectives. Our backgrounds within health care means that we may see health issues in a specific way and take certain issues for granted, some of which it is easier to become aware of than others. However, this could also mean that we were well positioned to do this study in the sense that we had appropriate background knowledge of the field [54].

## Results

An important context to the results was the collaborative quality improvement. Despite some initial skepticism, the QIC became a well-appreciated arena for the sharing of experiences and reflections on how to improve care. The participants experienced that the QIC targeted issues that were central to their everyday practice and meeting others with similar work experience and challenges was stimulating. However, the participants experienced various degrees of support to carry on the work and implement improvement measures in their own workplace.

In the following, the results are presented as three interrelated themes. In different ways, the themes shed light on how the participants understood and used the WMTY question in the quest to make transitional care for older people more patient-centered.

### WMTY is a complex process that needs to be framed competently

Overall, the participants understood WMTY as not “just a question” and more as a complex process that required competence to be used in practice. At meetings, it became clear that the participants perceived the problem of implementing WMTY into practice as mainly a problem of HCPs’ lack of competence. The ability to understand how, when, and where to use WMTY “correctly” was seen as important.

There was a shared perception among the participants that WMTY was a difficult and sometimes even unpleasant question to ask. The participants attached several descriptions to WMTY, such as “too big,” “dangerous,” and “soaring.” During an interview, one participant said the following:

*It is a very huge question. Maybe your life has changed tremendously. Maybe you have gotten a cancer diagnosis, then you come home, and we wonder, “What matters to you now?”*

The negative perceptions seemed to revolve around the openness of the WMTY question. Subsequently, the participants focused on framing and contextualizing the question to facilitate implementation into practice. Each improvement team tried to apply WMTY in different ways and to systematize the use of the question. Many expressed that this process was chaotic, despite valuable peer support from the QIC. It became apparent that the participants used WMTY both in a literal sense and as a symbol for a new approach to patients. During learning sessions in the QIC, both these ways of perceiving the question were emphasized, and this seemed to create uncertainty regarding how to apply the question into practice. One example of how the question was applied in a more literal sense was the decision to include the WMTY question as standard text in the hospital’s electronic discharge report. This would enable the goals of the patient to be handed over from one setting to the other. Other teams discussed how they could incorporate the question into multidisciplinary meetings by adding a column on the patient-board designated as WMTY, placing it alongside the columns for other vital parameters such as fall risk assessment or vital sign measurements. However, WMTY could also be interpreted too literally, for example, when the question was asked as the first question on arrival to the hospital. Participants gave examples of situations in which it was found challenging, or even inappropriate, to ask the question straight out. When WMTY was problematized during the QIC meetings, it was emphasized that the question was not meant to be literal but rather an expression of a new approach. One participant said the following:

*The workers in the home care services problematize the question and say that it is far too big. One has to try to make the question less dangerous; how shall we plan ahead? What matters to you? What do you want in the future? So, I think it needs to be rendered less dangerous. And if your focus is on user involvement, then it really is just a part of a conversation and a larger approach.*

Another participant expressed a more open and literal understanding of the question:

*Many think that they ask the “What matters to you?” question, but they maybe really do not ask the question. It is a difficult question to ask. So, one might express oneself in a way that the patient might interpret it as if “Do I want to go home, or do I want to go to intermediate care?” In a way, we must dare to ask it just as it is, “What matters to you?”*

Furthermore, the participants discussed the risk of building “castles in the air” with WMTY. There was a perception that if WMTY was not framed according to their context, the patient could get the impression that anything was possible. One participant thought it was difficult for the patients to be asked what matters if their needs could not be fulfilled. During an interview, another participant said the following:

*Now I am speaking for myself, I am afraid to ask for example, “What matters to you?” Yes, what will come out of it? Because it is pretty comprehensive. I have to direct it toward something then. I can ask, “What matters to you?” and think “In relation to what?” In relation to health? In relation to living at home? In relation to what? So, for example, yesterday I thought, “How should I ask that question to someone then? If I do not know myself what I can promise?” If you understand me? If I am to ask someone “What matters to you?” then in a way I have to direct it toward them living longer at home …*

This illustrates a central finding. The participants’ discomfort when asking WMTY also seemed to be grounded in an uncertainty about their responsibility to fulfill the needs and preferences of patients within limited resources. Asking WMTY seemed to create insecurity and a need for clarification about what was their responsibility as HCPs and what was their mandate in a more political sense. This was illustrated by the following quote:

*What is it really that the municipality wants us to be occupied with? Should we be giving necessary health services such as nutrition and personal hygiene and all that, or is the goal to find what matters to you? Because then, where is the borderline here? What is it really we should be doing? What is the responsibility of this municipality? Are we supposed to go shopping with Peter because that is what he wants?*

The participants’ response to this uncertainty was to frame WMTY to make it fit within the limits of what they believed they could offer.

The need to frame WMTY also seemed to be grounded in the experience that older patients had a hard time with and needed guidance in answering WMTY. There was a general perception and experience that the patients found it difficult to relate to the question and that framing the question by either changing it or adding to it gave more meaning. In particular, the timing of the question became a salient issue. These perceptions of asking WMTY appeared to be influenced by the transitional care context, one characterized by relatively short-term encounters with older patients during demanding situations, such as admittance to hospital and after discharge to the home.

Hence, the HCPs had to understand how to interpret and frame the WMTY question depending on the circumstances. In some situations, WMTY could be used successfully as it is, but in other situations, WMTY was more suggestive of a new attitude and way of communicating and working with the patients. Thus, WMTY seemed to become a way to talk about the principles of patient-centeredness. What the participants referred to as “contextualizing” the WMTY question could be interpreted as the participants trying to take some control over a complex new way of working, which they perceived as challenging both for themselves and for older patients.

Two different approaches emerged when the participants talked about asking WMTY, which involved two different roles that seemed to originate from two different understandings of the purpose of asking WMTY and the responsibility that comes with it.

### Framing WMTY as a functional approach

Most participants connected WMTY to goal setting, especially the goal of the older person regaining or maintaining functional independence to be able to cope at home. The introduction of the WMTY questionnaire as a way of approaching and measuring patient- centeredness in the QIC seemed to influence the participants’ focus on goal setting as a practical approach for implementing WMTY. Also, the participants understood WMTY as a way of making the patient take more responsibility for their own life and health situation and to become more actively involved in their own care. Hence, the purpose of WMTY in transitional care was to enable older people to cope with everyday life, especially to be able to return home and stay home with as little help as possible after a period of illness. In this way, WMTY seemed closely linked to the goal of increased efficiency, which was prominent in the quality improvement setting. When asked about the purpose of WMTY, one participant answered the following:

*It is to somewhat unlock the resources and self-care in the patients so that they can manage, to say it in a bit of an ugly way, so that it becomes cheaper for health care.*

One important aspect of the functional approach was that certain goals were seen as more important than others. Goals concerning physical function connected to being physically independent and managing at home, such as mobility and dressing, where highly valued and encouraged. Goals that had less to do with physical function, such as spare time activities or more existential needs, were mostly perceived as futile. Furthermore, WMTY was often limited to being a question of where the patient should transition to next: home or to an intermediate institution before coming home. The importance of avoiding nursing home admissions was highly emphasized.

From the functional approach, the role as an “elicitor of suitable functional goals” was derived. Most participants emphasized that the WMTY question was about encouraging the older patient to come up with answers or goals that the HCPs could do something about; they emphasized goals that generated concrete tasks and that made it possible for them to use their practical competence in some way. During an interview, one team leader said that they were not “pleased with” the answers that the patients gave to the WMTY question because it did not give them enough of a base to structure their work from. One participant elaborated by stating the following:

*Many are quick to answer, also when they need home care, what matters to you? Ok, I can answer my son matters to me, my wife matters to me, walking in the mountains matters to me, but our task is not your son, your wife, or the mountains, but what matters? So even here, you have to try to sort things out a bit.*

Thus, contextualizing WMTY into a functional approach also reflected an orientation toward tasks. The goals of the patient were accepted only in so far they were relevant to how the HCPs worked and deemed realistic to achieve within their available resources. The participants were also concerned with whether the goals would be realistic or unrealistic in terms of what the older person could manage at home. WMTY could turn into a process of “digging for” goals that would fit these criteria. This implied leading the patient toward certain suitable ways of answering WMTY. What emerged as a kind of ideal situation was when WMTY could be useful both for the HCP and for the patient. This appeared to be understood as a negotiation process, where the goals of the patient and those of the HCP would ultimately coincide, illustrated by this quote:

*Well, it is like this, if one thinks about the issue of independence, which we focus a lot on: “Ok, you should be able to walk the stairs, for example, on your own so that you can manage going to the store by yourself.” But for the user, what mattered the most was managing to walk to the community social club, and then you see that, in a way, for him to be able to go to the social club, he might also have to be able to walk the stairs, so then the wishes, the goals kind of coincide.*

The older patients’ ability to reflect on and verbally express goals in a coherent way was a central challenge and dilemma. The older patients and their caregivers had to be willing and able to talk about managing at home in the stressful context of transitional care, and this emerged as an important challenge.

Many participants emphasized that asking WMTY in this way was about contributing to the efficiency of services and decreasing the need for services, thus prioritizing basic functional needs. There seemed to be a collectively shared perception of the need for streamlining and cost saving to meet future challenges in care for older people, and this was also an important message at the learning sessions in the QIC. One participant reasoned the following:

*Ok, should we prioritize buying new winter shoes when there is 20 degrees minus outside or should we buy a ball gown? Ok, if you cannot afford both, then you have to prioritize, and then, you have to do it on what matters and that which we agree upon in health care and the political environment and hopefully among the users, that we have kind of a mutual understanding of it. And then, I think, what has to do with function and covering basic needs that is very pertinent for most people, I think so.*

### Framing WMTY as a relational approach

In addition to the functional approach, which was more apparent, a relational approach was established from the analysis. Most participants also perceived that WMTY was about more than functional goals and managing at home. A participant put it as the following:

*It shouldn’t just be about, I think, wishing to be able to walk or be able to walk independently and go home, but it should maybe also incorporate existential issues. And I feel kind of in a way that this is something we carry with us from before, but now, it is being opened to encompassing even a bit more.*

The relational approach involved getting to know the patient, being open for conversation, and establishing dialogue. Seeing the whole person and not just the patient was emphasized. Some participants experienced that asking WMTY led to the discovery of new things about the patient, things that they would not otherwise have gotten to know through a traditional assessment. This sometimes involved an element of surprise. The participants tried to see things from the patient’s perspective and understood that patients might have different views and preferences than what they had assumed. One participant said that WMTY is different because the answers you get are different. Another participant recalled a situation in which she had asked a patient WMTY:

*But yes, that cancer-patient (...) she also had something else that mattered to her, and that was to be able to get outside in the sun. And I don’t know, in a way, if I would have gotten that information from her if I hadn’t asked that question. Then, at the same time, I am thinking, oh lord, how important wouldn’t that be for someone who might not have long left to live!*

Thus, there seemed to be an understanding that WMTY was about giving the patient a personal voice. This involved letting go of one’s own agenda and being more open to understanding the patients’ perspectives. This also involved the understanding that what was important from a medical perspective might not be what mattered to the patient:

*So maybe for me treating the patient it is very important that I treat the patient’s heart failure, among other things, by giving him diuretics. However, for the patient, it is bothersome if he needs to go to the toilet and urinate every five minutes ( … ) or if he needs to get up in the evening or at night to urinate.*

There was a perception that HCPs often take for granted that they know what matters to the patient and that asking WMTY could make them more aware of their preconceptions about the patient. One participant said the following:

*And we would very much like to hear the user’s voice and take it into account. And to get to know what matters to the user, we actually have to ask, if not, we are just guessing. And then, it is easy that we guess based on what matters to us instead.*

Furthermore, there was a duality in the relational approach because it seemed to be understood both as a purpose in its own right and a way of motivating the patient so that it might be easier to involve the patient in a plan for how to manage at home after discharge. One participant said:

*It is clear, that question, that we have focused on it, “What matters to you?” I think that it is very nice, because it is about us becoming even more conscious about cooperating with the users, because it might take just a small thing for them to be able to stay at home.*

From the relational approach, the role as “open-minded facilitator of the personal voice” was derived. In this role, the HCP would ask WMTY without leading it toward functional goals, discharge planning, or the home situation. There was no right or wrong answer, and the use of relational skills was highlighted. The HCP should then ideally use this information to improve care in some way; often, this would be about “small things,” such as taking a hospitalized patient out for a stroll in the sun. This seemed to open up for identifying with the patient and establishing a different kind of relationship. Furthermore, this approach was understood as a way of preventing unnecessary treatments and assessments, ensuring that what was being done was in line with the patient’s wishes.

Many of the participants experienced that older people found it difficult to voice what mattered to them. Spending time with the patient and cooperating with family and informal caregivers was seen as ways of overcoming this challenge.

Although many participants expressed an understanding that WMTY was about more than functional goals, the issues of not having enough resources to engage fully in the relational approach came up. The participants were concerned with not having enough time to sit down, have a conversation about *what matters*, and have the time to listen and respond to the patient’s answers:

*In the setting and organization that we have in health care now in Norway, when it comes to somatic illness, I am talking about the somatic hospitals, you do not have the opportunity for all this, in the sense that it becomes one hundred percent incorporated. Because we lack time, because it is maybe a conversation that takes forty-five minutes to complete, so that you get the essence of what matters to the patient.*

To sum up, there seemed to be a tension between the two approaches toward WMTY, which were based on two different understandings of the WMTY question and the responsibility that comes with asking it. The functional approach seemed to imply a limitation of the question as being about goals connected to how to cope and physically function as independently as possible at home and seeing the patient as a patient. Hence, the HCPs responsibility was also limited to these domains of care. The relational approach seemed to imply asking WMTY more openly and being more open to seeing the “whole person,” not just their functional goals. This implied assuming responsibility for providing care within the larger context of the person’s life. For both approaches, the participants highlighted the influences of contextual constraints.

## Discussion

The main findings of this study are that HCPs working to make transitional care more patient- centered found WMTY challenging in several ways. Asking WMTY in practice was not simply a matter of whether or not HCPs ask the question but of *how* and *when* they ask the question, how they frame it, what answers they expect, and how they believe they can respond to the answers. The participants emphasized that the question was a simplification of an underlying complex process that needed to be framed competently. There was a tension between WMTY as a relational approach, where the question was interpreted more openly, and a functional approach, where the question was directed at certain functional goals related to the older person getting home and staying home.

### What matters to the patient or what matters to the professional?

Our findings reflect a general insecurity regarding the professional competence and responsibilities that a patient-centered approach requires [37, 38]. Furthermore they point to a tension between the personal goals of the patient, which are related to the smaller and larger things in the patient’s’ life, and the professionals’ goals, which necessarily are more disease-and function-centered. An important cause of the tension between the two approaches might lie in different ways of defining or limiting the HCP's responsibility in relation to working within the context of the older patient’s life. HCPs are inevitably drawn toward tasks and issues that concern disease and function, as this is where their expertise lies [14, 34]. If HCPs perceive that their responsibility is limited to improving the physical function and independence of the patient, then the WMTY question might be framed accordingly. However, patient-centered goal setting aims toward including multiple dimensions of the older patient’s life [14, 24, 25], and effective chronic care management seems to necessitate moving care into the context of the person’s life project [29, 33]. In the relational approach, there seemed to be a perception that HCPs are responsible for more than disease and function. The personal dimension was actualized to a larger degree. By asking WMTY with an open attitude, the participants realized that what mattered to the patient was different and could be more important than what mattered to them. By letting go of their own agendas to a larger extent, they could avoid putting time and energy into the things that did not necessarily matter to the patient. Previous research shows that this may reduce the burden on the patient and lead to care that is more effective [39, 55, 56]. Consistent with goal setting theory [28] this also seemed to be aimed at motivating the patient. Furthermore, the functional approach sheds light on the necessary and complex challenges of translating the often fuzzy and changeable goals of the patient into professional action. In line with this, Naldemirci et al. [25] emphasize that a central challenge in PCC is to overcome the distance between the patient’s everyday knowledge and a biomedical view of the patient’s problems. Our results indicated that when aiming to translate what matters to the patient into professional goals, HCPs risk framing and limiting the WMTY question to the extent that the underlying intent of the question is lost. There seemed to be a fine line between negotiating goals with the patient and persuading the patient to accept professional goals. The uncertainty regarding *the limits* of the HCPs' new responsibility in relation to asking WMTY was thus an important finding and is in line with previous research [45, 57]. A central critique of the patient-centered approach is that it represent a blurring of responsibility, not only for professionals, but also in relation to the increased responsibility given to the patients themselves [57].

Based on the concept of a patient-centered goal process [14, 58], the new responsibility that comes with asking WMTY is accepting and placing the patient’s goals on top of the goal hierarchy as well as to base the translation of these goals on the knowledge of the person behind the patient. Thus, a possible resolution to the tension in the findings is to incorporate the functional approach into the relational approach. Henceforth, there is a need to deliberate and clarify both HCPs’ and older patients’ responsibilities in relation to working within the frame of the patient’s life project and what can be expected of health care for chronically ill older people.

### Professional competence- understanding what matters and doing what matters

The participants experienced a lack of competence in relation to asking WMTY. With regards to competence, the relational approach, where the HCP had the role as a facilitator of personal voice aligns with an emphasis on relational competence [41] as well as the compassion dimension of WMTY. In other words, the emphasis was on *understanding* what matters*.* The functional approach contained elements of task orientation and on *doing*, aligning with an emphasis on action competence [41] and the goal-setting dimension of WMTY.

A salient issue pertaining to the need for competence to ask WMTY is that older people do not always express their needs and preferences as clear goals that are easily translatable into professional goals and actions [14, 25]. People often express what matters to them through more subtle forms of communication, *enacting* rather than verbally expressing what matters to them in everyday life [25, 59]. Consistent with previous research [8, 9, 14, 25, 60], the participants experienced that it was sometimes challenging for older patients in transition to respond to WMTY. To understand what mattered to the patient a certain amount of time was required. Hence, the participants’ focus on appropriate framing and timing of the question may point to the importance of relational competence [41] when eliciting what matters to older patients. The need for relational competence also seems to align with having a “WMTY conversation” which is promoted by the IHI [18, 23].

Furthermore, concerning, the functional approach, or *doing* what matters, the participants discussed the risk of understanding the WMTY question too simplistically or superficially. McCormack suggested that when HCPs base their communication on a too simplistic understanding of PCC, they may come to think that it is about *fulfilling* all the needs and preferences of the patient rather than listening, reflecting, and responding in a reflected manner [61]. The uncertainty and discomfort around the WMTY question may be understood based on this. In line with previous research [60–62], our findings imply that deciding upon what professional actions to take in relation to what matters to the patient is an interactive process characterized by negotiation. Our findings might thus encourage a reflection upon what competences HCPs currently use and what competences they may need to develop further when engaging in this process with older chronically ill people.

### What matters to you, or what matters to the system?

Working conditions and the larger political agenda for older people seemed to influence the participants’ framing of the WMTY question significantly. Hence, HCPs do not only have to overcome the distance between personal and professional goals, but also between what matters to the patient and system level goals. These findings are in line with current conceptualizations of PCC as a complex, multifactorial approach operating at micro, meso and macro levels [37, 38]. In line with previous research, [8, 9], the participants in this study feared unrealistic goals and not being able to meet the needs of the older patients within the current contextual constraints of transitional care. They tried to make the WMTY question fit with what they believed they could offer and avoid building “castles in the air”. Our findings thus suggest that the influence of conflicting goals and constraints could have led the participants to approach WMTY in a task-oriented manner. These findings align with previous research claiming that constraints at the system level may reinforce a paternalistic and task-oriented care approach [8, 25, 45, 63–65]. This implies that without appropriate changes at the macro- and meso level, there is a risk that WMTY may become just another catchy phrase, reinforcing an already existing practice, thus operating as “the emperor’s new clothes.”

### Strengths and limitations

A strength of the current article is that it sheds light on how HCPs make sense of the WMTY question in practice, something that should be highly relevant given its widespread use. The trustworthiness of this study is strengthened by the fact that several authors were involved in the analysis allowing for taken for granted assumptions to be uncovered and challenged. Only the first author was immersed in the field and critical discussion with “outsiders” provided a necessary balance of closeness and analytical distance to the material [54]. Furthermore, the writing of an analytical memo that served as a” decision trail” [66] as accounted for in the method section, hopefully strengthened the transparency and trustworthiness of the study. Use of quotes in the results section, and the use of theory and previous research to substantiate results in the discussion, should also strengthen the trustworthiness [54].

A limitation might be that we collected and analyzed data on “talk” about PCC practice; we did not observe practice itself. On the other side, the complementary data collection of interviews and observations of meetings should strengthen the trustworthiness of our findings [54]. In addition, following the participants over a prolonged period of time and in multiple contexts allowed for nuancing and clarification of findings as an ongoing process [54]. However, the choice to analyze based on commonalities across settings rather than local variations, inevitably led to some loss of nuances in the results [51].

With regards to the transferability of results, we strove to gain access to a broad representation of perspectives during data collection [54]. However, the fact that all the participants were chosen by their superior to participate in the QIC leads us to assume that they might have had certain common traits such as a general positive attitude toward quality improvement and thus practice changes. This might have influenced the results and should be considered when contemplating transferability to other contexts [54]. Nonetheless, we hope that our results may widen, or nuance, existing repertoires for understanding the implementation of PCC through the WMTY question.

### Practice implications

Our analysis implies that there is considerable uncertainty among HCPs regarding asking WMTY and the new roles, responsibilities and competence inherent in a patient-centered approach. HCPs should reflect on and become aware of which approaches and competences they use in relation to the WMTY question. We suggest that trying to incorporate the more familiar functional approach within the relational approach seems to be a way to move forward. A QIC may support HCPs in this effort by focusing more on concrete patient-centered practice models in addition to the WMTY questionnaire. There are a number of practice models within PCC, which can facilitate patient-centered goal setting with older chronically ill people [18, 23, 58, 67]. All in all this may reduce the insecurities of HCPs and may facilitate a move toward a more patient-centered practice.

In transitional care there needs to be agreement across all settings and professions on setting the patient’s goal on top of the prioritization list. We found that powerful contextual factors at macro- and meso level ultimately shaped the use of the WMTY question strongly. Leaders and organizations have to make room for reflection around the conditions HCPs have for asking older chronically ill people *what matters* and what responsibility it implies. Judging by our results, engaging in a QIC could be a fruitful arena for such reflection.

## Conclusion

WMTY is apparently a simple question, but using it in everyday practice is a complex process, which requires professional competence. In transitional care practice, we saw a tension between a relational and a functional approach to WMTY. The discussion suggests a possible resolution of this tension by incorporating the functional approach into the relational approach so that care is given within the frame of the patient’s life project. The importance of appropriate contextual conditions is also highlighted.

When seen in terms of a patient-centered goal setting process, our results shed light on HCPs’ challenges of eliciting the personal goals of the older patient, accepting those goals for what they are and finally yet importantly transferring the patient’s goals into realistic, rational and manageable professional goals. A relevant topic for future research is to look closer into the challenges of the WMTY process for example by observing relevant practice situations with older people.

## Supplementary information

**Additional file 1.** Thematic interview guide

## Data Availability

The data generated and analyzed in this study will not be shared, as the data collection approval for the study requires that the data is accessible only to the researchers.

## Abbreviations

HCP: Health care provider
WMTY: “What matters to you?”
PCC: Patient- or person-centered care

## Footnotes

1 When used synonymously, the abbreviation PCC is used; when used separately, the appropriate term is used.

## References

[1] Kastner M, Hayden L, Wong G, Lai Y, Makarski J, Treister V (2019). Underlying mechanisms of complex interventions addressing the care of older adults with multimorbidity: a realist review. BMJ Open.

[2] Coleman EA, Williams MV (2007). Executing high-quality care transitions: a call to do it right. J Hosp Med.

[3] Coleman EA, Boult C (2003). Improving the quality of transitional care for persons with complex care needs. J Am Geriatr Soc.

[4] Naylor MD (2006). Transitional care: a critical dimension of the home healthcare quality agenda. J Healthc Qual.

[5] Norwegian Ministry of Health and Care services. The Coordination Reform — Proper treatment – at the right place and right time. Report No. 47 to the Storting (2008-2009). Oslo, Norway. 2008.

[6] Dahl U, Johnsen R, Saetre R, Steinsbekk A (2015). The influence of an intermediate care hospital on health care utilization among elderly patients--a retrospective comparative cohort study. BMC Health Serv Res.

[7] Storm M, Siemsen IM, Laugaland K, Dyrstad DN, Aase K (2014). Quality in transitional care of the elderly: key challenges and relevant improvement measures. Int J Integr Care.

[8] Dyrstad DN, Testad I, Aase K, Storm M (2015). A review of the literature on patient participation in transitions of the elderly. Cogn Tech Work.

[9] Allen J, Hutchinson AM, Brown R, Livingston PM (2017). User experience and care integration in transitional Care for Older People from Hospital to home: a meta-synthesis. Qual Health Res.

[10] Kvael LAH, Debesay J, Bye A, Bergland A. Health-care professionals' experiences of patient participation among older patients in intermediate care-at the intersection between profession, market and bureaucracy. Health Expect. 2019. 10.1111/hex.12896 PMC6803410 31127681

[11] Lilleheie I, Debesay J, Bye A, Bergland A (2019). Experiences of elderly patients regarding participation in their hospital discharge: a qualitative metasummary. BMJ Open.

[12] Coffey A, Mulcahy H, Savage E, Fitzgerald S, Bradley C, Benefield L (2017). Transitional care interventions: relevance for nursing in the community. Public Health Nurs.

[13] Håland E, Røsstad T, Osmundsen TC (2015). Care pathways as boundary objects between primary and secondary care: experiences from Norwegian home care services. Health.

[14] Berntsen GK, Gammon D, Steinsbekk A, Salamonsen A, Foss N, Ruland C (2015). How do we deal with multiple goals for care within an individual patient trajectory? A document content analysis of health service research papers on goals for care. BMJ Open.

[15] Dyrstad DN, Testad I, Storm M (2015). Older patients' participation in hospital admissions through the emergency department: an interview study of healthcare professionals. BMC Health Serv Res.

[16] Schouten LM, Hulscher ME, van Everdingen JJ, Huijsman R, Grol RP (2008). Evidence for the impact of quality improvement collaboratives: systematic review. Bmj.

[17] Barry MJ, Edgman-Levitan S (2012). Shared decision making--pinnacle of patient-centered care. N Engl J Med.

[18] IHI.org. What matters 2019 [Available from: http://www.ihi.org/Topics/WhatMatters/Pages/default.aspx. Accessed 12 July 2019.

[19] Norwegian Ministry of Health and Care services. A full life - all your life — A Quality Reform for Older Persons (White paper no 15 (2017–18)) Oslo, Norway. 2017.

[20] Minister of Health, Bent Høie's speech; The patient's helathcare services (pasientens helsetjeneste). [press release]. Oslo, Norway. 2014.

[21] Bisognano M (2012). A simple lesson plan for patient-centered care. Hosp Health Netw.

[22] Bisognano M. Ask patients: 'what matters to you?'. AHA News. 2012;48(17).

[23] Kebede S. Ask patients" What matters to you?" rather than" What's the matter?". BMJ. 2016;354. 10.1136/bmj.i4045 27449399

[24] Hakansson Eklund J, Holmstrom IK, Kumlin T, Kaminsky E, Skoglund K, Hoglander J (2019). "Same same or different?" a review of reviews of person-centered and patient-centered care. Patient Educ Couns.

[25] Naldemirci O, Lydahl D, Britten N, Elam M, Moore L, Wolf A (2018). Tenacious assumptions of person-centred care? Exploring tensions and variations in practice. Health (London).

[26] Moore L, Britten N, Lydahl D, Naldemirci O, Elam M, Wolf A (2017). Barriers and facilitators to the implementation of person-centred care in different healthcare contexts. Scand J Caring Sci.

[27] Reuben DB, Tinetti ME (2012). Goal-oriented patient care--an alternative health outcomes paradigm. N Engl J Med.

[28] Locke EA, Latham GP (2002). Building a practically useful theory of goal setting and task motivation. A 35-year odyssey. Am Psychol.

[29] Fried TR, Street RL Jr, Cohen AB. Chronic disease decision making and "what matters Most". J Am Geriatr Soc. 2020. 10.1111/jgs.16371 PMC7197748 32043559

[30] Kruglanski AW, Shah JY, Fishbach A, Friedman R, Chun WY, Sleeth-Keppler D. A theory of goal systems. Advances in Experimental Social Psychology. 2002;34:331–78.

[31] Austin JT, Vancouver JB (1996). Goal constructs in psychology: structure, process, and content. Psychol Bull.

[32] Institute of Medicine (U.S.). Committee on Quality of Health Care in America. Crossing the quality chasm : a new health system for the 21st century. Washington, D.C.: National Academy Press; 2001. xx, 337 p. p.

[33] Hansen F, Berntsen GKR, Salamonsen A (2018). "What matters to you?" A longitudinal qualitative study of Norwegian patients' perspectives on their pathways with colorectal cancer. Int J Qual Stud Health Well Being.

[34] Harding E, Wait S, Scrutton J (2015). The state of play in person-centred care.

[35] Naldemirci O, Wolf A, Elam M, Lydahl D, Moore L, Britten N (2017). Deliberate and emergent strategies for implementing person-centred care: a qualitative interview study with researchers, professionals and patients. BMC Health Serv Res.

[36] McCormack B (2003). A conceptual framework for person-centred practice with older people. Int J Nurs Pract.

[37] Mik-Meyer N The power of citizens and professionals in welfare encounters : the influence of bureaucracy, market and psychology. Manchester: Manchester University Press; 2017. xii, 158 pages p.

[38] McCormack B, McCance TV (2006). Development of a framework for person-centred nursing. J Adv Nurs.

[39] Dwamena F, Holmes-Rovner M, Gaulden CM, Jorgenson S, Sadigh G, Sikorskii A (2012). Interventions for providers to promote a patient-centred approach in clinical consultations. Cochrane Database Syst Rev.

[40] Epstein RM, Hundert EM (2002). Defining and assessing professional competence. JAMA.

[41] Røkenes OH, Hanssen P-H. Bære eller briste. Kommunikasjon og relasjon i arbeid med mennesker [sink or swim. Communication and relation in work with people]. 3rd ed. Bergen: Fagbokforlaget.; 2012. 311 p.

[42] Spitzberg BH, Hecht ML (2006). A component model of relational competence. Hum Commun Res.

[43] Krohne K, Torres S, Slettebø Å, Bergland A (2013). Individualizing standardized tests: physiotherapists’ and occupational therapists’ test practices in a geriatric setting. Qual Health Res.

[44] Nygren P (2004). Handlingskompetanse: om profesjonelle personer [action competence: on professional persons].

[45] Olsen CF, Bergland A, Debesay J, Bye A, Langaas AG. Striking a balance: health care providers' experiences with home-based, patient-centered care for older people-a meta-synthesis of qualitative studies. Patient Educ Couns. 2019. 10.1016/j.pec.2019.05.017 31160128

[46] Berger PL, Luckmann T (1991). The social construction of reality: a treatise in the sociology of knowledge: penguin Uk.

[47] Kilo CM (1998). A framework for collaborative improvement: lessons from the Institute for Healthcare Improvement's breakthrough series. Qual Manag Health Care.

[48] Stratford P, Gill C, Westaway M, Binkley J (1995). Assessing disability and change on individual patients: a report of a patient specific measure. Physiother Can.

[49] Malterud K (2001). Qualitative research: standards, challenges, and guidelines. Lancet.

[50] Malterud K, Siersma VD, Guassora AD (2016). Sample size in qualitative interview studies: guided by information power. Qual Health Res.

[51] Braun V, Clarke V (2006). Using thematic analysis in psychology. Qual Res Psychol.

[52] Braun V, Clarke V (2013). Successful qualitative research: a practical guide for beginners: sage.

[53] Denzin NK, Lincoln YS (2011). The sage handbook of qualitative research: sage.

[54] Moen K, Middelthon A-L. Qualitative research methods. Laake, P, Benestad, HB, Olsen, BR (Eds) (2015) Research in medical and biological sciences- From Planning and Preparation to Grant Application and Publication: Elsevier. Academic press; 2015. p. 321–78.

[55] Coulter A, Entwistle VA, Eccles A, Ryan S, Shepperd S, Perera R. Personalised care planning for adults with chronic or long-term health conditions. Cochrane Database Syst Rev. 2015;(3):Cd010523. 10.1002/14651858.CD010523.pub2 PMC6486144 25733495

[56] Tinetti ME, Naik AD, Dindo L, Costello DM, Esterson J, Geda M (2019). Association of Patient Priorities–Aligned Decision-Making with Patient Outcomes and Ambulatory Health Care Burden among Older Adults with Multiple Chronic Conditions: a nonrandomized clinical trial. JAMA Intern Med.

[57] Mik-Meyer N (2017). The power of citizens and professionals in welfare encounters: the influence of bureaucracy, market and psychology.

[58] Berntsen G, Hoyem A, Lettrem I, Ruland C, Rumpsfeld M, Gammon D (2018). A person-centered integrated care quality framework, based on a qualitative study of patients' evaluation of care in light of chronic care ideals. BMC Health Serv Res.

[59] Pols J (2005). Enacting appreciations: beyond the patient perspective. Health Care Anal.

[60] Jokstad K, Skovdahl K, Landmark BT, Haukelien H (2019). Ideal and reality; community healthcare professionals' experiences of user-involvement in reablement. Health Soc Care Community.

[61] McCormack B (2017). Negotiating partnerships with older people: a person centred approach: Routledge.

[62] Kvael LAH, Debesay J, Bye A, Bergland A. The dramaturgical act of positioning within family meetings: negotiation of Patients' participation in intermediate care services. Qual Health Res. 2019:1049732319873054. 10.1177/1049732319873054 31526100

[63] Sharp S, Mcallister M, Broadbent M (2018). The tension between person centred and task focused care in an acute surgical setting: a critical ethnography. Collegian.

[64] Hestevik CH, Molin M, Debesay J, Bergland A, Bye A (2019). Older persons' experiences of adapting to daily life at home after hospital discharge: a qualitative metasummary. BMC Health Serv Res.

[65] Johannessen AK, Werner A, Steihaug S (2014). Work in an intermediate unit: balancing between relational, practical and moral care. J Clin Nurs.

[66] Noble H, Smith J (2015). Issues of validity and reliability in qualitative research. Evid Based Nurs.

[67] Britten N, Moore L, Lydahl D, Naldemirci O, Elam M, Wolf A (2017). Elaboration of the Gothenburg model of person-centred care. Health Expect.

