# Supplementary material for: What matters when asking, “what matters to you?” — perceptions and experiences of health care providers on involving older people in transitional care
Source: BMC Health Serv Res. 2020 Apr 16;20:317. doi: 10.1186/s12913-020-05150-4 (PMC7164237; doi:10.1186/s12913-020-05150-4)
Supplement: Supplementary file 1 — Additional file 1. Thematic interview guide [file 12913_2020_5150_MOESM1_ESM.docx]

**Additional file 1**

**Thematic interview guide**

| **Introduction**  **Background for entering this quality improvement collaborative**  **Perceptions of being a part of this quality improvement collaborative** |
| --- |
| **Describe work in improvement team so far**  **Current improvements measures**  **Describe/give examples of experiences and challenges** |
| **Perceptions of current challenges in transitional care for the older chronically ill**  **Give example of a dream scenario of a care/patient pathway for the older chronically ill**  **Give example of a worst case scenario of a care/patient pathway for the older chronically ill** |
| **What matters to you?**  **General perceptions of WMTY question**  **How the improvement team has approached it**  **Practical application of WMTY question in respective workplace, ask for concrete examples, as well as possible challenges or dilemmas**  **How WMTY might relate to the QIC’s goal of attitudinal and cultural change**  **Relationship between WMTY and the older patients’/users’ choice in the care/patient pathway**  **Resources in relation to asking WMTY**  **(Interviewer brings and shows the WMTY questionnaire to facilitate recall)** |
| **Perceptions of the care pathway development process**  **Perceptions of the care pathway model with checklists**  **Perceptions and experiences with the use of checklist**  **(Interviewer brings example of cp model and checklist to facilitate recall)** |
| **Interviewer enquires about issues or occurrences during observations of meetings in the QIC** |
| **Final open comments or opinions on the topics discussed** |
